# GFAP-NpHR mediated optogenetic inhibition of trigeminal nucleus caudalis attenuates hypersensitive behaviors and thalamic discharge attributed to infraorbital nerve constriction injury

**DOI:** 10.1186/s10194-023-01669-z

**Published:** 2023-10-11

**Authors:** Elina KC, Jaisan Islam, Hyong Kyu Kim, Young Seok Park

**Affiliations:** 1 Program in Neuroscience, Department of Medicine, College of Medicine, Chungbuk National University, Cheongju, 28644 Republic of Korea; 2 Department of Medicine and Microbiology, College of Medicine, Chungbuk National University, Cheongju, 28644 Republic of Korea; 3 Department of Neurosurgery, Chungbuk National University Hospital, Cheongju, 28644 Republic of Korea

**Keywords:** Astrocytes, Medullary dorsal horn, Optogenetics, Trigeminal neuralgia

## Abstract

The significance of hyperactive astrocytes in neuropathic pain is crucial. However, the association between medullary astrocytes and trigeminal neuralgia (TN)-related pain processing is unclear. Here, we examined how optogenetic inhibition of medullary astrocytes in the trigeminal nucleus caudalis (TNC) regulates pain hypersensitivity in an infraorbital nerve (ION) constricted TN model. We used adult Sprague Dawley rats subjected to infraorbital nerve (ION) constriction to mimic TN symptoms, with naive and sham rats serving as controls. For in vivo optogenetic manipulations, rats stereotaxically received AAV8-GFAP-eNpHR3.0-mCherry or AAV8-GFAP-mCherry at the trigeminal nucleus caudalis (TNC). Open field, von Frey, air puff, and acetone tests measured pain behavioral flexibility. In vivo thalamic recordings were obtained simultaneously with optogenetic manipulation in the TNC. Orofacial hyperalgesia and thalamic hyperexcitability were both accompanied by medullary astrocyte hyperactivity, marked by upregulated GFAP. The yellow laser-driven inhibition of TNC astrocytes markedly improved behavioral responses and regulated thalamic neuronal responses. Halorhodopsin-mediated inhibition in medullary astrocytes may modify the nociceptive input transmitted through the trigeminothalamic tract and pain perception. Taken together, these findings imply that this subpopulation in the TNC and its thalamic connections play a significant role in regulating the trigeminal pain circuitry, which might aid in the identification of new therapeutic measures in TN management.

**Graphical Abstract:**

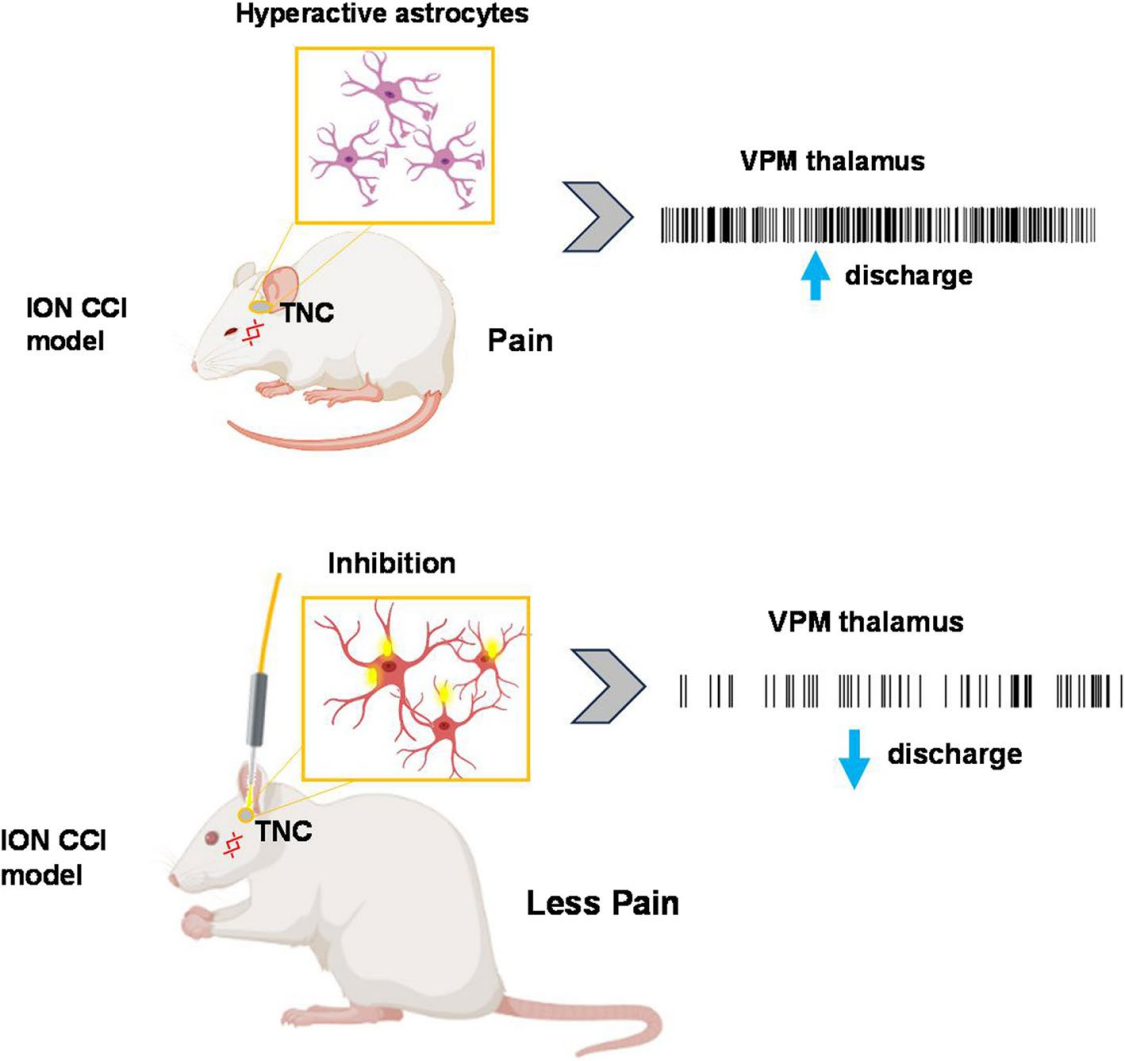

**Supplementary Information:**

The online version contains supplementary material available at 10.1186/s10194-023-01669-z.

## Introduction

According to the International Association for the Study of Pain, trigeminal neuralgia (TN) is a form of orofacial neuropathic pain that corresponds to one or more branches of the trigeminal nerve [1]. The condition is marked by abrupt, intense, stabbing pains that are typically unilateral, reoccur frequently, and significantly diminish the overall quality of life for affected individuals [2]. Contrary to the spinal dorsal horn, the trigeminal nucleus caudalis (TNC) serves as the primary central node within the trigeminal nociceptive pathway, situated in the brain stem nucleus. The TNC principally receives nociceptive afferent signals originating from the orofacial region [3]. Nociceptive neurons within the trigeminal ganglion (TG) acquire TN cues and convey them to the TNC [4]. The pathophysiology of trigeminal pain involves a complex interplay of biological interactions among central nervous system (CNS) resident cells such as neurons, astrocytes, and microglia, like mechanisms observed in spinal neuropathic pain [5]. The most prevalent glial cells in the CNS, astrocytes, are crucial for maintaining processes notably homeostasis and synapse development [6]. Multiple astrocyte modifications induced by nerve damage contribute significantly to the amplification of pain signals [7]. A growing body of research suggests that chronic pathological conditions [8–10] such as TN [11] are associated with the presence of hyperactivated astrocytes, particularly implicated in the modulation of pain perception. Despite reports of glial cell activation within TNC following trigeminal nerve injury, direct empirical investigation regarding the involvement of TNC astrocytes in TN pathophysiology remains limited [12].

Many research findings have shown that altering signaling pathways in astrocytes presents a promising avenue for the development of innovative approaches in the treatment of chronic pain [13–15]. However, a wide range of existing approaches encounter challenges, including multiple adverse effects and inadequate cell specificity [16]. In recent years, the exploration of neuropathic pain mechanisms, encompassing conditions like TN, has been significantly expedited by the emergence of optogenetics, which enables the selective activation or inhibition of target cells within neural circuits [17]. With the advance of optogenetic techniques, it has recently become feasible to manipulate astrocyte activity in vivo precisely by employing a specific promoter of astrocytes paired with light sensitive opsin [18]. Not long ago, a group of researchers first reported the use of optogenetics to stimulate spinal astrocytes in live rats and suggested that suppressing astrocyte activation could hold immense therapeutic promise for treating chronic pain [19]. In the context of trigeminal pain, pharmacological studies have indicated that hyperactive astroglia play a significant role in modulating nociceptive responses in the trigeminal spinal nucleus following trigeminal nerve injury [20, 21]. Despite these findings, no study to date has acknowledged an astrocyte-specific approach employing optogenetic tools to regulate astrocyte activity in the TNC in a trigeminal pain model. This highlights the novelty and potential significance of our research endeavor.

In the present study, we employed an optogenetic approach aiming to manipulate the astrocyte population in the TNC to investigate whether the selective silencing of this set of cells can ameliorate pain-like behavior induced by chronic constriction of the infraorbital nerve. As the trigeminal thalamic pathway is strongly implicated in the sensory-discriminative aspects of TN, we further examined the effect of this inhibition in ventral posterior medial thalamic discharge. Our results support the previous studies which suggests that the hyperactivity of astrocytes in the TNC paralleled orofacial hypersensitive behaviors and aberrant thalamic discharge. Our hypothesis is that disrupting the ventral trigeminothalamic tract (VTT) could potentially alleviate pain, suggesting a specific pathway through which astrocyte modulation could impact pain processing. We further examined the expression of subtype of purinergic receptors (P2X3) and neuropeptide (CGRP), both of which are implicated in the pathophysiology of TN.

## Materials and methods

### Animals

A total of 51 female Sprague Dawley rats (8 weeks old, weighing 200–250 g) were purchased from Koatech Bio, South Korea. The rats were housed in ventilated cages with autoclaved wood chip bedding under standard settings, with a temperature of 23 °C, a 12-h cycle of light and dark, and a humidity level of 30%. Except during experimental sessions, free access to standard rodent food and water was supplied. Between 9:00 am and 6:00 pm, surgical procedures and behavioral testing were carried out.

#### Surgical procedures

The chronic constriction of the infraorbital nerve (ION), as previously described, generated the TN model [22]. The rats were randomly assigned into two groups-TN (*n* = 23) and sham (*n* = 23). General anesthesia was used for all surgical operations, and intraperitoneal injections of 9 mg/kg xylazine (Rompun®, Bayer AG, Leverkusen, Germany) and 15 mg/kg Zoletil (Zoletil50®, Virbac Laboratories, Carros, France) were administered. Briefly, the infraorbital nerve was exposed through a tiny incision above the eye area. Using Dumont forceps for blunt dissection, the ION was isolated from the nearby connective tissue and muscle. The left ION bundle was wrapped in two loose ligations with a gap of 2 mm, and the incision was closed with 3–0 silk sutures. The contralateral sides were left intact in every rat. Rats that underwent sham surgery did not have their nerves clipped. The entire surgical process was performed in an aseptic manner. Following surgery, one rat was kept in each cage for recovery, with sterile bedding. In Additional file 1 (Figure S1), the experimental timetable is illustrated.

#### AAV virus injection for optogenetic manipulation

We employed adeno-associated virus (AAV) infused with a red fluorescent protein (mCherry) and astrocyte promoter (GFAP) to specifically target the astrocyte population in the TNC. We used AAV8-GFAP-eNpHR3.0-mCherry, which targets the expression of halorhodopsin in the astrocyte populations, for optogenetic investigations. AAV8-GFAP-mcherry was used as control or null virus. The KIST viral facility (Korea Institute of Science and Technology, Seoul, Republic of Korea) provided the viruses. The optogenetic and null viruses exhibited concentrations of 6.93 × 10^13^ GC/ml and 2.01 × 10^14^ GC/ml, respectively.

Following ION constriction or sham surgery, anesthetized rats were mounted on a stereotaxic platform for virus injection into our designated brainstem region (TNC). The rat's body temperature was maintained at 36 °C with the aid of a heating pad, and each group received the appropriate viral injection following the previous protocol [23, 24]. The coordinates were defined as mediolateral (ML) from the midline, anterior–posterior (AP) from the bregma, and dorsoventral (DV) from the brain's pial surface. Briefly, the cranial adjacent skin was slit to expose the skull, and a hole of 1 mm diameter was drilled into the skull (-13.8 mm AP and -2.5 mm ML, -7.8 mm DV). Using the Paxinos rat brain atlas [25] and previous publications [26, 27], the injection site coordinates for the TNC was determined and the tip of the Hamilton syringe was moved slowly into the TNC to prevent excessive tissue damage. Using an automated microsyringe pump (KD Scientific Legato® 130 Syringe Pump, Harvard Apparatus, Holliston, MA, USA), the optogenetic virus was administered to TN or sham rats (designated TN/eNpHR + or sham/eNpHR + further) while a null virus (designated TN/eNpHR- or sham/eNpHR- further) was administered to the other group. The infusion rate was 0.2 μL/min, and the injection volume per animal was 2 μL. For proper virus diffusion and to avoid leakage, the needle was left in the target for five minutes. The incision site was stitched once the needle was carefully withdrawn. The animal was removed from the stereotaxic frame and put back inside its home cage.

#### Behavioral assessment

A researcher who was unaware of the interventions performed the behavioral analysis. Each rat was handled and accustomed to the experimenter's hand and the testing area before each test. A day before (baseline test) and weekly for three weeks following ION constriction or sham treatment, the behavioral responses in response to mechanical and thermal stimulation were assessed.

For the open field test (OFT), each rat was kept in a black arena-sized 70 cm × 70 cm × 30 cm and videotaped for 5 min with an overhead camera that was positioned vertically 1 m above the test field. The number of explored areas and mobility rate were then determined from the video recordings utilizing automated open-source executable software (ToxTrac) for image-based tracking [28]. Post OFT, the mechanical hypersensitivity and allodynia of the orofacial region were assessed using the graded series of von Frey (VF) filaments (bending forces: 0.4, 1.4, 6, 8,15 g, 26 g) and the air puff test (APT), respectively. In both the ipsilateral and contralateral vibrissal pads, the filaments were evaluated in ascending order of force. A quick head retraction and/or an attack/escape reaction were counted as the responses. The mechanical response threshold was defined as the smallest force required to cause at least one of these responses when applied through von Frey filaments [29]. APT is based on the notion that when the injured orofacial region is exposed to continuous air puffs of varying pressures, the face withdraws. After 10 consecutive trials of continuous air-puff pressure (4 s long and 10 s apart) were applied to the trigeminal territory, withdrawal behavioral reactions such as an escape from air-puff or aggressive behaviors like biting were witnessed [30, 31]. The air puff pressure and intervals were regulated by a pneumatic pump module (BH2 system, Harvard Apparatus, USA). A 26-gauge metal tube (length: 10 cm) held at a 90-degree angle to the skin was used to deliver air puffs. For air puffs, the pressure cutoff was 40 psi (pounds per square inch). A considerable drop in the pressure threshold relative to baseline readings was used to characterize mechanical allodynia. A couple of drops of cold acetone were applied to the vibrissal pad region to measure the extent of the cold allodynia subsequently developed after the injury. Within the first two minutes following acetone delivery, nocifensive behavior such as rubbing or scratching on the ipsilateral whisker pad was noted [32]. Other than asymmetric orofacial grooming, body rubbing was not included. The number of responses was added up after each of the three behavioral tests, which were spaced out by five minutes.

#### In vivo electrophysiology and fiber optic cannula implantation

After three weeks of virus injection, in vivo recording and fiber cannula insertion were carried out for optimum viral expression. Extracellular recordings from anesthetized rats (15 mg/kg Zoletil® and 9 mg/kg xylazine as the anesthetic dose) were conducted in a Faraday cage in a quiet setting with low lighting. Rats were positioned inside a stereotaxic device, and a craniotomy was carried out above the thalamic ventral posteromedial (VPM) region (AP, 3.5 mm; ML, 2.8 mm, DV, -6.0 mm). A fiber optic cannula (MFC_200/230–0.48_###_ZF2.5_A45, Doric Lenses, Quebec City, Quebec, Canada) was stereotaxically implanted 0.2 mm dorsal to the viral injection site. Dental cement and superbond (Ortho-jet Pound Package, Lang Dental, Wheeling, IL, USA) were used to secure the cannula to the rat skull. A quartz-insulated carbon microelectrode (E1011-20, Carbostar-1, Kation Scientific, Minneapolis, MN, USA) was then placed contralaterally (DV = -5.6 mm) to the cannula implantation site. The attached screws served as anchors for the ground and reference wires that connected the electrode to the electronic interface board (EIB-36, Neuralynx, USA). A Cheetah Acquisition System (Neuralynx, USA) was implemented to acquire VPM thalamic output through the EIB-36's 36-channel headstage and preamplifiers. In a Digital Lynx SX data-acquisition system (Neuralynx, Bozeman, USA), neuronal signal bands were filtered at 0.9–6 kHz and the sampling frequency was set to 32 kHz. With simultaneous light-off and light-on instances in the TNC, acute recordings (spontaneous and evoked triggered with 10 g von Frey filament provocation on the ipsilateral whisker pad) from the VPM were obtained. Without supplementary anesthesia, the recording period for each rat lasted approximately two hours. Before the subsequent experiment, the animals were placed back in their home cage for a seven-day recovery period. Spikesort 3D software was used to further analyze the raw data to identify and isolate clusters. Waveforms with separable clusters and distinctive properties were referred to as single units and exported to NeuroExplorer (version 5, Nex Technologies, Colorado Springs, USA), which offers a full range of spike train analysis tools, including rate histograms, rasters, and burst analysis.

#### In vivo optogenetic manipulations in free-moving animals

Animals were taken to the behavior assessment area after a week of complying with the electrophysiological experiment and habituated for 30 min before the start of the test session. We used a diode-pumped solid-state 589-nm yellow DPSS laser (model: YL589T3-010FC, Shanghai Laser & Optics Century Co., Ltd., Shanghai, China) coupled with a monofiber optic patch chord that was placed over the cranial window in vivo for full-field stimulation to drive cells that were expressing mCherry and NpHR. We used a minimal irradiance of 0.4 mW/mm2 for 100% spiking inhibition on eNpHR3.0 expressing neurons during steady 593 nm laser illumination. A waveform generator (Keysight 33511B-CFG001, Santa Rosa, CA, USA) was employed to control the delivery of a 5 min yellow light pulse (593 nm, 8 mW, constant). When TNC astrocytes were silenced for five minutes using continuous yellow light, behavioral tests (OFT, VF, AT, and AP) were conducted. In both instances, a tethered fiber system and associated equipment were attached.

#### Immunostaining and imaging

Animals transfected with AAV8-GFAP-eNpHR3.0-mCherry or AAV8-GFAP-mCherry were optically stimulated with continuous yellow light for 10 min followed by deep anesthesia with a Zoletil/xylazine mix. PBS and 4% paraformaldehyde (PFA) were transcardially administered to rats. The brain and TG were removed, preserved in 4% PFA overnight, and then submerged in a 30% sucrose solution. The tissues were cryo-frozen and embedded in a compound with optimum cutting temperature (OCT; Tissue Tek®, Sakura, USA). Tissue  sections (20 μm) were obtained from the cryostat for immunostaining. Sections were fixed in acetone for 10 min and blocked at room temperature for 1 h in 10% normal goat serum. Slides were further incubated in mouse anti-GFAP (1:250, ab68428, Abcam), mouse anti-c-fos (1:1000, ab208942, Abcam), mouse anti-Iba1 (1: 100, ab283319, Abcam), and mouse anti-CGRP (1:50, ab81887, Abcam) overnight at 4 °C. Sections were washed and incubated for 2 h at room temperature with appropriate secondary antibodies (Alexa Fluor 488, ab150077, ab150113, Abcam). Mounting medium with DAPI (H-2000, Vectashield®, Vector Laboratories Inc., Burlingame, CA 94010) was used and coverslipped. The slides were imaged using a fluorescence microscope with cellSens Standard (Olympus Corp., Tokyo, Japan) or OlyVia 2.4 software (Olympus Corp., Tokyo, Japan). For each immunohistochemical staining and quantitative analysis, three coronal sections including the TNC and TG, were chosen. Quantifying the intensity in the red channel allowed for the identification of mCherry fluorescence. The mean fluorescence in a reference region close to the injection site was used to define and remove the background fluorescence from the image. By deducting the area of the selected cell*mean background fluorescence values from the integrated density, the fluorescent intensity or corrected total cell fluorescence was determined [33]. ImageJ software (National Institutes of Health, MD, USA) was used to compare and quantify the mean fluorescence intensity between groups.

To determine P2X3 expression in the TNC, chromogenic immunohistochemistry was used. The recombinant anti-P2X3 antibody (ab300493, Abcam) was incubated overnight at a 1:50 dilution with acetone-fixed frozen sections after preincubation for one hour in 2.5% normal horse serum. Following washing, slices were treated with biotinylated anti-mouse/rabbit IgG and then subjected to an avidin–biotin HRP procedure using an ABC kit (PK-7200, Vector Laboratories, Burlingame, CA) and diaminobenzidine (DAB substrate kit, SK-4100, Vector Laboratories, Burlingame, CA). Using the immunohistochemical image analysis toolbox of ImageJ software, the color detection method was used to quantify P2X3.

#### Statistical analysis

The data are presented as the mean ± standard deviation (SD). The sample sizes were established using G*Power (version 3.1.9.4, Germany) and prior experimental considerations. Rats exhibiting insufficient viral expression were excluded from the study. Two-tailed Mann–Whitney test, Welch’s t-test or an ordinary two-way analysis of variance (ANOVA), followed by Tukey’s or Sidak’s post hoc test based on the experimental terms, were used to conduct statistical analyses of the experiments to determine the significance. *P < 0.05, **P < 0.01, ***P < 0.001 and ****P < 0.0001 were used to identify significant differences. For the statistical evaluations, GraphPad Prism (version 9.1.0, Inc., San Diego, CA, USA) was utilized.

## Results

### Validation of astrocyte selective viral-mediated transduction in the TNC

**Fig. 1:**
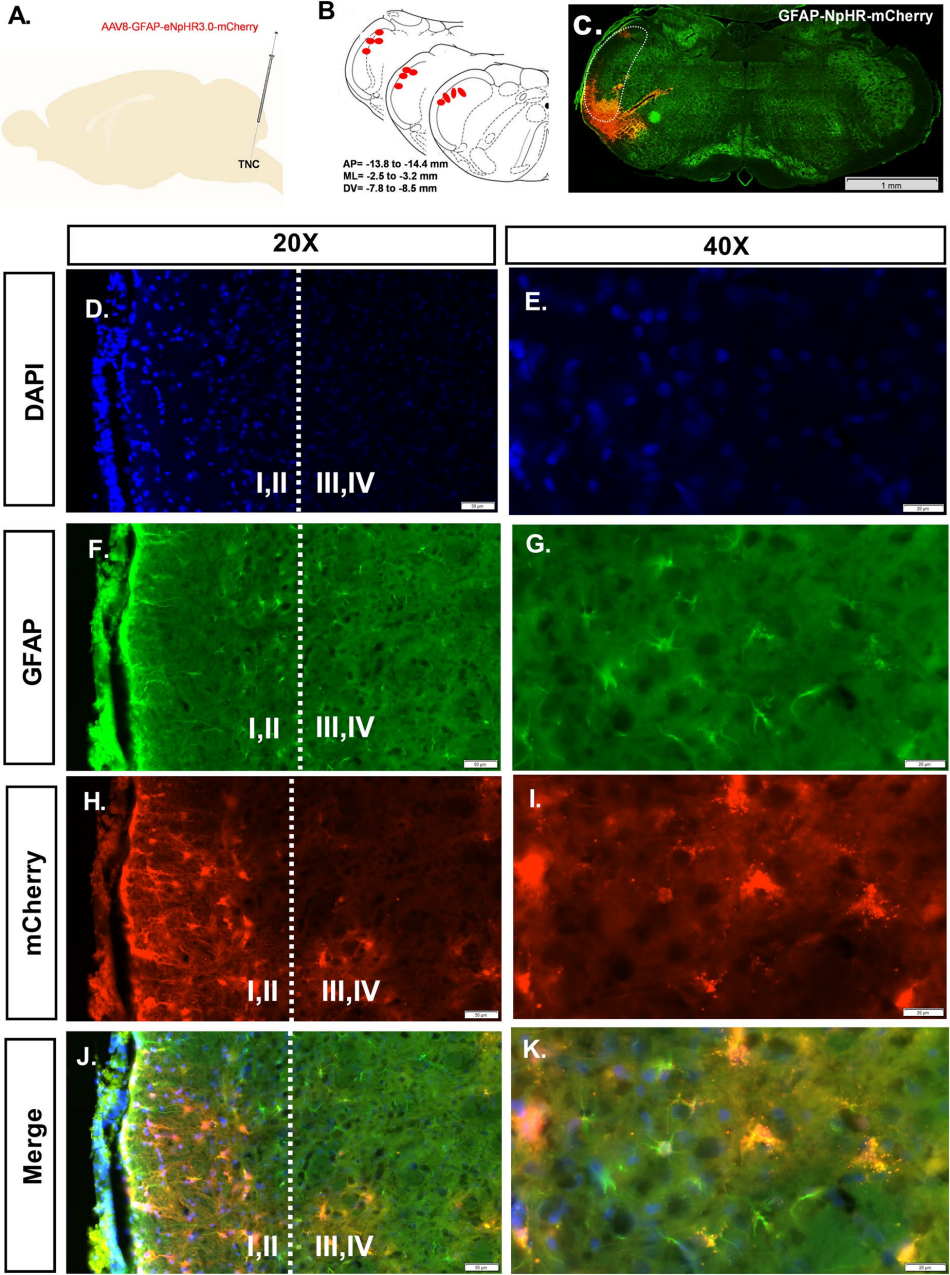
Virus expression in the TNC. **A** Schematic drawing of the site for virus injection **B** Coronal section of TNC, red dots showing the virus localization site **C** Representative fluorescent image showing the coronal section of TNC and virus localization, Scale = 1 mm. **D**, **E** DAPI (**F**, **G**) GFAP (**H**, **I**) eNpHR-mCherry (**J**, **K**) Merge images for colocalization of optogenetic virus expression in the TNC, Scales = 50 μm and 20 μm. Dashed white lines marked the laminar divisions in TNC

In this investigation, we used the inhibitory opsin eNpHR3.0. The expression of each construct was regulated by the astrocyte-specific promoter-GFAP, and opsin was coupled with red fluorescent protein-mCherry. All constructs were packaged into AAV8 vectors. We virally transduced TNC astrocytes using procedures that have been previously outlined. Viral injection was performed unilaterally in the TNC areas, and six weeks later, histological examinations were performed. As shown by most GFAP-positive astrocytes that were virally transduced, we confirmed the astrocyte selectivity of GFAP-dependent vectors following the earlier research (Fig. 1). To assess the efficiency of AAV virus transfection, we compared the mCherry fluorescence intensity in the TNC between the groups. Insignificant differences between the groups were found (Additional file 1, Figure S2).

### Trigeminal neuralgia upregulates GFAP expression and induces hypersensitivity

**Fig. 2:**
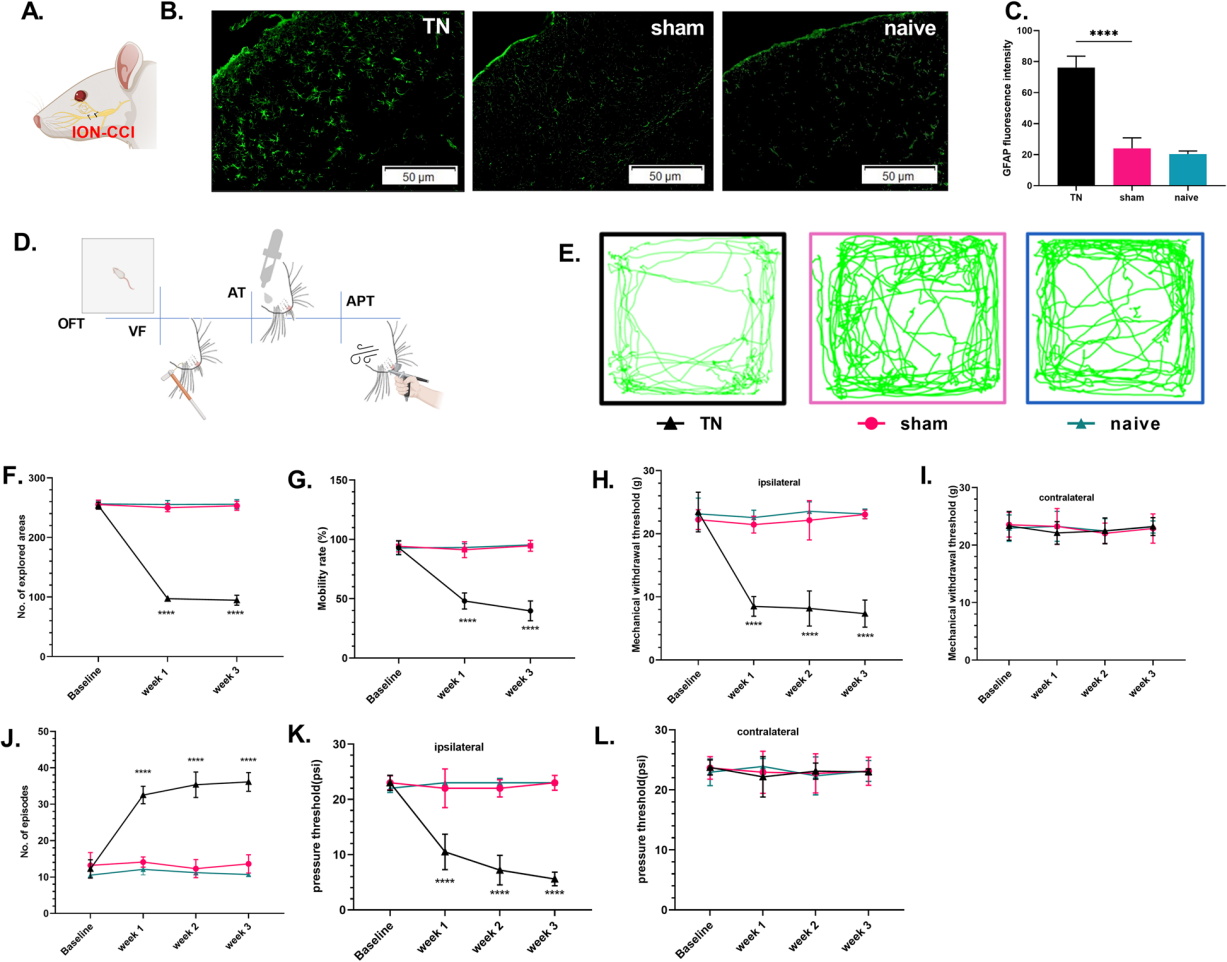
Infraorbital nerve constriction induced hyperalgesia and upregulated GFAP expression in the TNC (**A**) Schematic diagram of infraorbital nerve constriction in rats. **B** Immunofluorescence staining showed that GFAP fluorescence intensity was significantly increased in the TNC of the TN group compared to that of the sham and naïve groups. Scale bar = 50 μm. **C** Quantification of GFAP immunostaining in the TNC of different groups. Data are presented as the mean ± SD, *n* = 5/group, two tailed Welch’s t-test, ****p < 0.0001 compared with the sham group. **D** Pictorial diagram of the behavior test for pain assessment (**E**) Representative trajectory of TN, sham and naïve groups in the OFT. **F** Number of explored areas and (**G**) mobility rate (in percentage) in each group before (baseline) and after (week 1 and week 3) surgery. **H**–**L** The mechanical pain threshold in the von Frey filament test in ipsilateral and contralateral whisker pads. **J** The thermal pain threshold. **K**-**L** The pressure threshold in air puff test in ipsilateral and contralateral whisker pads. ****, *p* < 0.0001, ordinary two-way ANOVA followed by Sidak’s multiple comparisons among groups. Data are displayed as the means ± SD, *n* = 20(TN), 20( sham), 5* (naïve)*

A study has reported that behavioral hyperalgesia reliably coexists with astrocytic activation, which can occur following trigeminal nerve injury [34]. Anti-GFAP immunostaining on activated astrocytes was evaluated between the groups. After three weeks of IONC, the number of astrocytes, as indicated by cells displaying GFAP labeling, was larger in the TNC of TN mice than in sham-operated and naive rats (Fig. 2A-C). To investigate the association of TN with behavioral hypersensitivity and quantify neuropathic pain in our IONC model, we performed the following behavioral tests at weekly intervals for three weeks following IONC: OFT, VF, AT, and APT (Fig. 2D). A transient decrease in the number of explored areas {F (2,126) = 633.6, *p* < 0.0001} and mobility rate {F (2,126) = 71.82, *p* < 0.0001} in the OFT test in TN rats was observed at 7 days after the surgery, indicating the anxious state of these rats. It should be noted that in the OFT, which is also often used as a test for depression-like behavior, a difference was observed between the TN and sham rats. No significant differences were observed between sham and naïve rats (Fig. 2E-G). The mechanical hypersensitivity of the TN rats was reflected by the decreased withdrawal threshold in the ipsilateral vibrissal pad during the VF {F (3,168) = 43.37, *p* < 0.0001} and APT {F (3,168) = 44.04, *p* < 0.0001} following injury in comparison to baseline assessment. No significant differences were noted in the contralateral pad (Fig. 2H, I, K, L). In addition, we found thermal hypersensitivity in TN rats with an increased number of episodes (Fig. 2J) in cold AT {F (3,168) = 69.48, *p* < 0.0001}. Since these behaviors were noticeably different from week one following the IONC surgery and persisted throughout the experiment, TN animals manifested overt hyperalgesia symptoms. Naive rats did not display these behaviors in the sham group of rats, who underwent comparable surgery but without nerve constriction. Accumulating evidence, including our results, suggests that astrocytes in the TNC are key players in hypersensitive behavior after nerve injury.

### GFAP/eNpHR mediated photoinhibition in the TNC modulates upregulated TNC astrocytes and VPM neuronal discharge

**Fig. 3:**
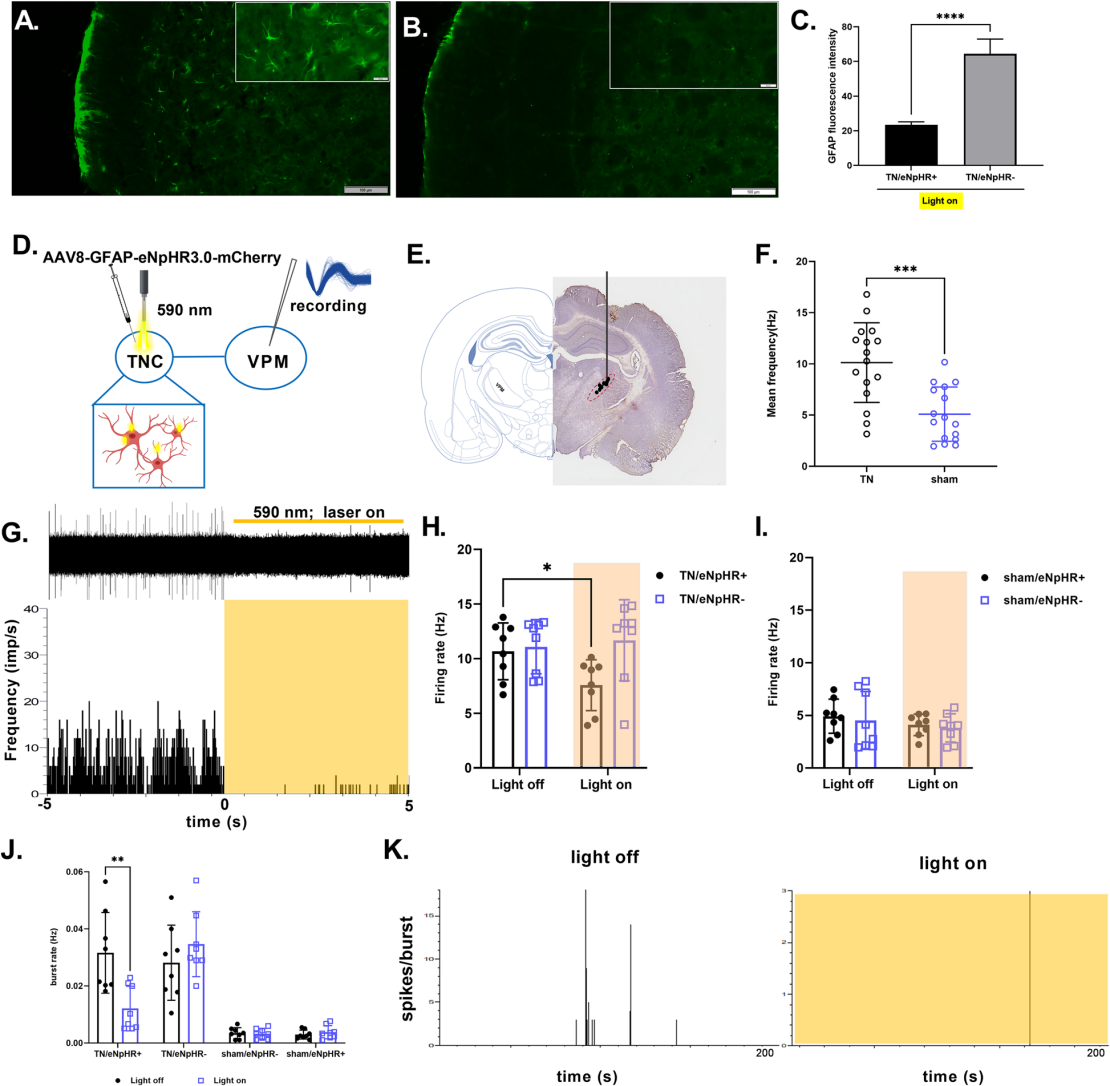
GFAP expression and VPM thalamic recording in response to optic inhibition of TNC astrocytes. **A**-**B** Representative fluorescent image of GFAP expression in the TNC of TN/eNpHR- (**A**) and TN/eNpHR + (**B**) rats in response to the optic inhibition of TNC astrocytes. Scale = 100 μm and 20 μm. **C** Quantification of GFAP immunostaining in TNC of the TN/eNpHR- and TN/eNpHR + groups in the yellow laser on condition, *n* = 6/group, two-tailed Welch’s t-test, ****p < 0.0001 compared with another group. **D** Schematic diagram of in vivo optogenetic manipulation of TNC astrocytes with a yellow laser (590 nm) in conjunction with the thalamic recording (**E**) Coronal section of the VPM thalamus showing the recording site. **F** Mean firing rate from the VPM thalamus between the TN and sham groups. Two-tailed unpaired t-test with Welch’s correction, *n* = 16/group, ***P = 0.0002 compared to sham (**G**) Representative thalamic discharge (lower one presented in frequency, impulse per second) with raw traces (upper) during light-off and yellow laser-on conditions in the TN/eNpHR + group. **H** Mean firing rate (presented in Hertz) from VPM thalamus of TN/eNpHR + and TN/eNpHR- groups during light off and on conditions. Two-way repeated-measures ANOVA with Sidak’s multiple comparisons test, *n* = 8/group, *P = 0.0380 compared to light off condition of TN/eNpHR + (**I**) Mean firing rate (presented in Hertz) from VPM thalamus of sham/eNpHR + and sham/eNpHR- groups during light off and on conditions. Two-way repeated-measures ANOVA with Sidak’s multiple comparisons tests, *n* = 8/group, not significant, *P* = 0.4800 compared to the light-off condition of sham/eNpHR + . **J** Burst frequency (presented in Hertz) comparison between TN (TN/eNpHR + and TN/eNpHR-) and sham (sham/eNpHR + and sham/eNpHR-) groups in response to light stimulation on and off in TNC. Two-way repeated-measures ANOVA with Sidak’s multiple comparisons tests, *n* = 8/group, **P = 0.0017 compared to light off condition of TN/eNpHR + . Data are presented as the mean ± SD. **K** Representative burst traces of TN/eNpHR + rat from the VPM thalamus during light-off and light-*on condition in TNC astrocytes*

Next, we asked whether GFAP/eNpHR mediated inhibition in the TNC can restore the upregulated the GFAP expression exhibited by activated astrocytes among TN/eNpHR + and TN/eNpHR- animals. As shown in Fig. 3A-C, GFAP immunoreactivity in the TNC was lower in TN/eNpHR + rats, suggesting the downregulation of activated astrocytes following optogenetic inhibition of TNC GFAP + cells.

Previous studies have suggested that changes in VPM neuronal activities seem likely to be a direct reflection of alterations in the primary afferent fibers of the constricted infraorbital nerve [33]. Likewise, we assessed VPM thalamic activity with in vivo electrophysiological recordings in the 4^th^ week after IONC. We found high frequency as well as burst firing in thalamic neurons in IONC rats, in comparison with sham and naïve rats suggesting neuroplastic changes in the thalamocortical circuits and producing abnormal sensory perception in the orofacial region (Fig. 3D-F).

Previous studies have revealed the projections of the caudal part of the spinal trigeminal nucleus to the contralateral somatosensory nuclei of the thalamus [35]. To address whether TN altered the excitability of VPM thalamic neurons and TNC astrocytes, electrophysiological characteristics were examined in the VPM thalamus in response to optogenetic modulation of TNC astrocytes. GFAP-eNpHR-mediated inhibition in the TNC significantly decreased the mean firing rate during the yellow light-on state in TN/eNpHR + rats (Fig. 3G). However, no difference was observed in the thalamic discharge of TN/eNpHR- rats in response to light stimulation. Likewise, there were no alterations in the sham animals under either light-on or light-off conditions (Fig. 3H, I). In addition to the mean firing rate, similar results were observed for burst firing. The burst rate and the percentage of spikes in the burst were significantly diminished in the TN/eNpHR + animals during light stimulation in TNC astrocytes (Fig. 3J, K; Additional file 1, Figure S3). We also observed that acute inhibition of astrocytes suppresses the neural activity in the TNC represented by decreased c-fos expression in TN/eNpHR + animals in response to yellow laser (Additional file 1, Figure S4). These results suggest that optogenetic modulation of TNC astrocytes mediated by enhanced halorhodopsin and astrocyte-specific promoter (GFAP) influence the neuronal activity of the orofacial sensory thalamus in TN animals.

### Optogenetic inhibition of TNC astrocytic activity reverses IONC-induced hyperalgesia and anxiety-like behaviors

**Fig. 4:**
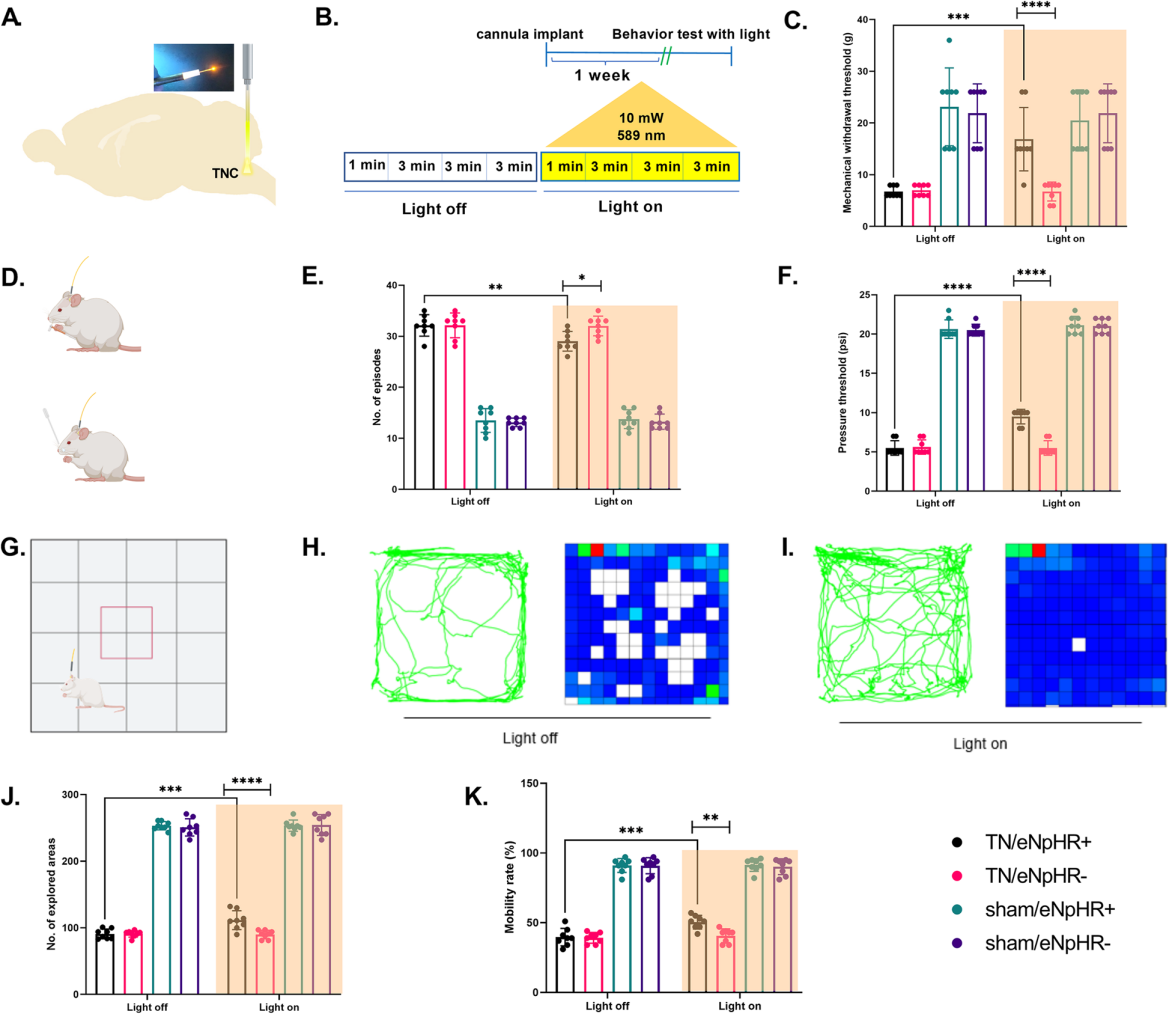
Optogenetic inhibition of TNC astrocytes alleviates pain induced by ION constriction. **A** Schematic diagram of the optic modulation site in rat brain. **B** Light stimulation parameters during the behavior test. **C** Optic inhibition of TNC astrocytes increased mechanical withdrawal threshold in ipsilateral whisker pad during the von Frey filament test in TN/eNpHR + rats. Data are presented as the mean ± SD. Two-way ordinary ANOVA with Sidak’s multiple comparisons tests, *n* = 8/group, ***P = 0.0006 compared to light off condition of TN/eNpHR + rats. Two-way ordinary ANOVA with Sidak’s multiple comparisons tests, *n* = 8/group, ****P < 0.0001 compared to light on condition between TN/eNpHR + and TN/eNpHR- rats (**D**) Schematic diagram of rats subjected to von Frey filaments (upper) and acetone (lower) tests with a tethered system for optogenetic manipulations. **E** Astrocyte-specific optic inhibition of TNC decreased the number of episodes during the acetone test for cold allodynia in TN/eNpHR + rats. Data are presented as the mean ± SD. Two-way ordinary ANOVA with Sidak’s multiple comparisons tests, *n* = 8/group, **P = 0.0075 compared to light off condition of TN/eNpHR + rats. Two-way ordinary ANOVA with Sidak’s multiple comparisons tests, *n* = 8/group, *P = 0.0160 compared to light on condition between TN/eNpHR + and TN/eNpHR- rats. **F** The pressure threshold during the air puff test was improved during TNC astrocyte inhibition with a yellow laser in TN/eNpHR + rats. Two-way ordinary ANOVA with Sidak’s multiple comparisons tests, *n* = 8/group, ****P < 0.0001 compared to light off condition of TN/eNpHR + rats. Two-way ordinary ANOVA with Sidak’s multiple comparisons tests, *n* = 8/group, ****P < 0.0001 compared to light on condition between TN/eNpHR + and TN/eNpHR- rats. **G** Schematic diagram of rat subjected to open field test with a tethered system for optogenetic intervention (**H**, **I**) Representative trajectory and exploration heatmap of TN/eNpHR + rats during light on and off conditions. **J**, **K** The number of explored areas (***P = 0.0007) and mobility rate (***P = 0.0004, expressed as a percentage) were considerably improved in TN/eNpHR + animals under light-on conditions compared to those under light-off conditions. No significant differences were observed in the other groups. Data are presented as the mean ± SD. Two-way ordinary ANOVA with Sidak’s multiple comparisons tests, *n* = 8/group, (explored areas, ****P < 0.0001; mobility rate, **P = 0.0012) compared to light on condition between TN/eNpHR + and TN/eNpHR- rats

To establish a direct link between the increased astrocytic activity of the TNC region and the hypersensitive behavior seen in IONC rats, we tested whether suppressing the astrocytes of the TNC would reverse the pain-like behaviors induced by IONC. Of primary interest is that IONC-induced pain in the TN/eNpHR + with yellow light stimulation group significantly increased the mechanical and thermal pain thresholds compared with those in the TN/eNpHR- and sham groups. The mechanical withdrawal threshold in VF was significantly improved during the light-on state (16.875 ± 6.128 g) in comparison to the light-off state (6.75 ± 1.035 g) in TN/eNpHR + rats. The pain scores in the TN/eNpHR- or sham groups (sham/eNpHR + and sham/eNpHR-) did not change with the same light stimulation without opsin expression indicating that light stimulation did not affect the pain sensation in sham or TN rats without eNpHR (Fig. 4A-C). Likewise, we observed similar results in APT and AT (Fig. 4D-F) with an increased threshold (light off: 5.5 ± 0.926 psi vs light on: 13.125 ± 2.588 psi) and a decline in the number of episodes (light off: 32.125 ± 2.100 vs light on: 20.75 ± 1.669). In the OFT, the number of explored areas and the mobility rate were significantly increased in the TN/eNpHR + group during light stimulation (Fig. 4G-K). This signifies that not only sensory pain, but also emotional attributes are influenced by optogenetic manipulations.

### Suppression of purinergic receptor P2X3 expression in the GFAP-transduced TNC

**Fig. 5:**
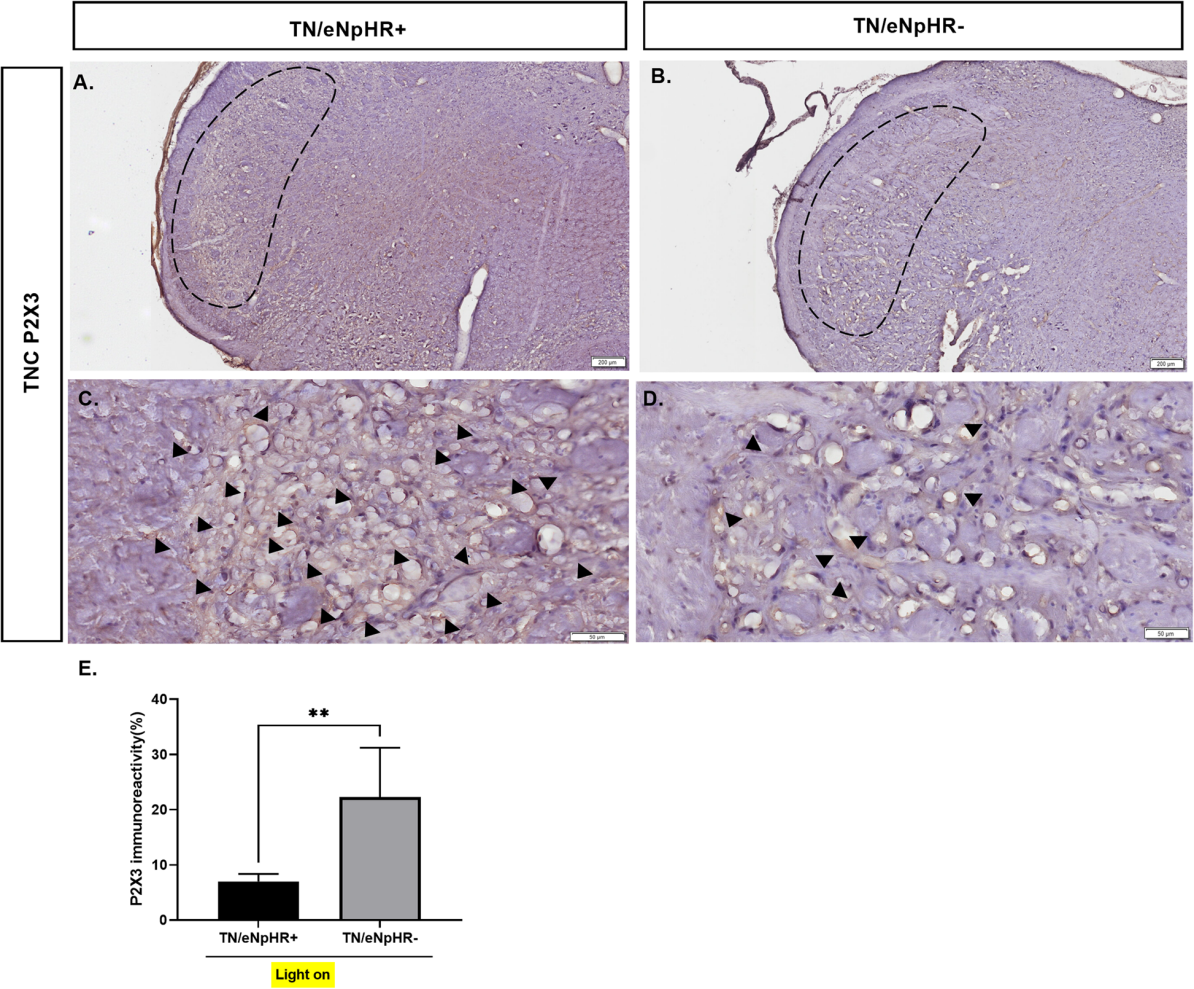
P2X3 immunoreactivity in the TNC in response to astrocyte-specific TNC optogenetic inhibition. Representative immunohistochemistry images showing P2X3 expression in the TNC of (**A**, **C**) TN/eNpHR + (**B**, **D**) TN/eNpHR- animals in response to astrocyte-specific TNC optogenetic inhibition. Black arrowheads represent the P2X3 expressed areas in the TNC. (**E**) Quantification of P2X3 immunoreactivity in the TNC of TN rats with optogenetic inhibition with a yellow laser. Data are presented as the mean ± SD. Two-tailed Mann–Whitney test, *n* = 6/group, **P = 0.0043 compared to another group

The purinergic receptor P2X_3_ is a nonselective cation channel, that is sensitive to ATP released during injury [36]. A significant increase in P2X_3_ immunoreactivity has been reported in brainstem astrocytes [37] as well as the TG of ION constricted rats [38]. Thus, we used immunohistochemistry to determine the level of P2X_3_ expression in the TNC. The preferential expression of P2X3 was significantly higher in the TN groups. We analyzed the effect of optic inhibition of TNC GFAP***-***transduced cells in P2X3 in TNC astrocytes. We found that the P2X3 expression level in the TNC was significantly decreased in the TN/eNpHR + group after illumination compared to the TN/eNpHR- group (Fig. 5).

### Optic inhibition of TNC astrocytes alters CGRP immunoreactivity and microglial expression

CGRP, a major neuropeptide in the trigeminal pain pathway [39] was examined by immunostaining and a high percentage of CGRP-immunoreactive neurons were expressed in TG of TN rats (Additional file 1, Figure S5). To test our hypothesis, we next asked whether TNC astrocyte inhibition can influence this neuropeptide in the TG, which is the predominant site of CGRP receptors [40]. We found that TN/eNpHR + rats illuminated with yellow light had less CGRP immunoreactivity in TG sensory neurons compared to TN/eNpHR- rats (Additional file 1, Figure S5). Moreover, we also examined microglial expression as microglia-astrocyte communication in pain hypersensitivity has been reported [41, 42]. Interestingly, our data revealed that there was a subsequent reduction in upregulated microglia, marked by Iba1 expression, following optogenetic inhibition in TN/eNpHR + rats (Additional file 1, Figure S5).

## Discussion

In the present study, we aimed to address the relationship of infraorbital nerve constriction and medullary astrocytes, both of which play pivotal roles in the pathogenesis of neuropathic pain associated with trigeminal neuralgia. To achieve this, we presented empirical evidence through the successful inhibition of astrocytes within trigeminal spinal caudalis via stereotaxic delivery of adeno-associated virus conjugated with enhanced halorhodopsin under the control of astrocyte-specific promoter GFAP. It has previously been reported that astrocytes in the medullary dorsal are involved in orofacial neuropathic pain following trigeminal nerve injury [21]. In our study, stereotaxic injection of the eNpHR virus enabled the expression of this opsin in medullary dorsal horn astrocytes, which were selectively inactivated by laser illumination through a cannula implanted on the site. Herein, we report a substantial and mechanistically significant alteration in the nociceptive behaviors of freely moving animals following the direct optogenetic inhibition of TNC astrocytes. We report heightened neurophysiological patterns of thalamic activity, suggesting that the silencing of TNC astrocytes exerts significant effects on the regulation of thalamic neuronal discharge as well. These findings extend our prior demonstrations illustrating the disruption of trigeminal-thalamic networks in trigeminal neuralgia and suggest the potential modulation of interrupted trigeminal-thalamic pain pathways through the selective targeting of TNC astrocytes.

Orofacial nociception may be triggered by distinct microcircuitry in the brainstem, but the precise mechanisms are still unclear. Within this context, the secondary neurons situated in the TNC play a pivotal role in the integration of nociceptive input from the TG neurons. Notably, the activity within the central nervous system related to the trigeminal nerve nociceptive pathway is reflected by the concurrent activity in the TNC and TG. The transmission of pain signals is significantly influenced by the activity of TNC, as underscored by previous research [43]. Abundant evidence has established a close link between astrocytes and TN. Trigeminal nerve injury has been related to orofacial hyperalgesia, heightened nociceptive responses, and a significant upsurge in both neuropeptide expression and astrocyte activity within the TNC, as evidenced by earlier investigations [44]. Important elements of atypical TN pain hypersensitivity are displayed in rodent models with chronic infraorbital nerve constriction, but relatively few researchers have discussed the involvement of astrocytes in the development of trigeminal dynamic mechanical allodynia in these models [11, 45]. These findings align with the results of the present study and corroborate similar experiments reporting hyperactivation of TNC astrocytes subsequent to TN injury induced by ION constriction. Contrary to the spinal cord, which synapses in the dorsal horn, the nociceptor afferents that innervate the head and mouth cavity enter the central nervous system via the trigeminal nerve. The trigeminal nerve enters the brainstem carrying nociceptive information and descends in the dorsolateral medulla to synapse onto the spinal trigeminal nucleus on the ipsilateral side [46]. In trigeminal nerve-constricted animals, there was a marked increase in astrocyte activation in the spinal trigeminal nucleus which could be associated with hyperalgesia. Earlier studies showed that inhibiting astrocyte activation can be an effective strategy to alleviate neuropathic pain [47–49]. However, direct and robust evidence supporting the role of TNC astrocyte function in TN has been limited. In this study, we adopted an optogenetic strategy and manipulated the activity of TNC astrocytes which provides extended evidence to existing studies. Our investigation aimed to determine whether the inhibition of medullary astrocyte activity, achieved through the use of inhibitory opsin conjugated with a specific promoter of astrocytes, could mitigate TN-induced hyperalgesic behaviors. Interestingly, our data revealed that astrocyte-specific optogenetic inhibition in CCI-induced TN rats attenuated behavioral hyperalgesia, as verified by increased pain thresholds in the VF, APT, and AT. We observed more pronounced improvements in mechanical sensitivity compared to thermal sensitivity upon suppression likely resulting from intricate and distinct neural pathways and mechanisms involved in processing these sensory modalities. Astrocytes can modulate synaptic transmission and neuronal activity in response to various factors, including pain signals. Their modulation might have different effects on the processing of mechanical versus thermal pain signals, depending on the specific neurotransmitter systems and receptors involved. Overall, our results suggests that behavioral hyperalgesia correlates with TNC astrocyte activation in a neuropathic pain model. Moreover, this astrocytic activation induced by TN could be optogenetically inhibited, suggesting that photoinhibited astrocytes have pain-alleviating effects. In addition to addressing hyperalgesia, the results of OFT also indicate that optogenetic inhibition of TNC astrocytes has an impact on anxiety-like behaviors in the study subjects. As this behavior may be secondary to the experience of pain, we did not further investigate other emotional aspects of pain after astrocytes were optogenetically inhibited. However, the observation of changes is intriguing and suggests a link between pain perception and emotional states. Furthermore, it has been reported that glial cell hyperactivity is produced faster in microglia than astroglia and that astroglial hyperactivity is longer lasting, suggesting that hyperactive astroglial cells in the TNC produced by the nerve injury may be involved in the maintenance of the trigeminal neuropathic pain state rather than the initiation of neuropathic pain [50].

There is substantial evidence that the dense projection fibers to the thalamus are sent by the spinal trigeminal nucleus, which contains the subnuclei oralis, interpolaris, and caudalis. Prior research has revealed that neurons within the TNC exhibit projections that extend to the contralateral thalamus [51, 52]. Modulation of the TNC influences its rostral projections, evoked transmitter release, and neuronal activity. Among these, the VPM thalamus is one of the important rostral projections from the TNC implicated in sensory discriminative features of orofacial pain [53]. The orofacial stimulus's pain quality, intensity, modality, and location are all mediated by this lateral pathway of the ascending nociceptive system that projects to the cortex [54]. In TN models, modulation of TNC astrocyte activity, particularly through the VTT, can play a role in modulating the neuronal activity of the orofacial sensory thalamus. The intensity and frequency of pain signals sent from the VTT to the VPM thalamus can be affected by the modulation of TNC neuronal activity by activated astrocytes, perhaps through ATP signaling. The in vivo recordings sought to ascertain whether TNC astrocyte-specific inhibition affects outputs from the TNC to the thalamus. Trigeminal nerve injury causes nociceptive neurons in the VPM to become hyperexcitable because the TNC transmits orofacial noxious impulses to the VPM and intralaminar thalamic nucleus [55]. Our findings corroborate prior studies demonstrating that ION constriction increased the VPM thalamic discharge in the TN groups. It is interesting to note that the optogenetic suppression of TNC astrocytes altered the increased thalamic discharge due to nerve injury. c-fos is a protein marker that is often used as an indicator of neural activity or neuronal activation. The acute inhibition of astrocytes reduces c-fos expression, indicating that astrocytes influence neuronal activation within the TNC. The observed modulation of VPM neuronal discharge suggests that astrocytic activity within the TNC has functional consequences for sensory processing. It is important for our understanding of pain and sensory perception to have insight into how astrocytes influence neuronal activity in sensory pathways.

The superficial laminae of the TNC serves as an important route of entry for noxious input from the orofacial region. After stimulating sensory nerve endings, TG neurons release CGRP, recognized key mediator responsible for conveying pain sensations to the superficial laminae of the TNC. The alteration of CGRP immunoreactivity suggests that TNC astrocytes may play a role in the regulation of pain signaling pathways. CGRP is often associated with pain modulation, so changes in its expression can have significant implications for pain perception. [56]. P2X3R, another significant purinergic receptor gated by ATP, is expressed in nociceptive primary afferents as well, aiding in the transmission of unpleasant mechanical stimuli to the central nervous system [38]. The interactions between astrocytes and neurons are believed to underlie the modulatory effects exerted by astrocytes, with certain chemicals, proteins, and their respective receptors presumed to be crucial in this process [57]. A review study raised the possibility that astrocyte-derived ATP and P2X receptors might alter the activity of glutamatergic synapses in the brain in a bidirectional manner [58]. A study reported a novel role for P2X3 in the fine astrocytic process in the mechanism of craniofacial neuropathic pain and preferential expression of P2X3 there as well. It also suggested that astrocytic mGluR5 may regulate astrocytic P2X3 expression. [37]. They also stated that astrocytes in the TNC exhibit functional P2X3Rs and that their density is elevated in response to trigeminal nerve damage, suggesting that they serve as an element in the signaling of craniofacial pain [59]. In our study, we examined the expression of CGRP and P2X3R in the TG and TNC, respectively. We noted that the optic inhibition of medullary astrocytes in neuropathic rats prompted diminished expression of CGRP and P2X3 in the neuropathic rats. This research suggests that TN enables hyperactivated astrocytes in the TNC, which are probably induced by ATP signaling through P2X3R activation. It is significant to highlight that in freely moving rats, laser stimulation of the astrocytic population in TNC expressing GFAP/eNpHR led to substantial and profound suppression of pain. The decrease in astrocytic activity and ATP release in TNC, as well as the altered VPM thalamic discharge, may be accountable for the pain-relieving response driven by the optogenetic suppression of TNC (GFAP/eNpHR expression in astrocytes). The propagation of calcium ion waves inside astrocytes is thought to be intrinsic to ATP release, and recent work suggests that similar communication may also take place between astrocytes and neurons [60]. Likewise, the impact on microglial expression indicates that TNC astrocytes may be involved in neuroinflammatory responses associated with trigeminal neuralgia. Our result also suggests a potential link between astrocytic activity and neuroinflammation in the condition consistent with other studies however additional research is crucial.

In conclusion, our study implemented optogenetic intervention in conjunction with electrophysiological and histological investigation to systematically elucidate the essential function of TNC astrocytes in TN. The present data consolidate the current understanding of this topic, and this study is the first to report that ION-induced hypersensitive behavior in a TN rat model could be regulated by TNC astrocytes modulation which also indirectly regulate the abberant thalamic discharge, especially through ventral trigeminothalamic tract. Astrocytes, by modulating synaptic transmission and possibly ATP release, can impact the excitability of TNC neurons and thus influence the transmission of pain signals to the thalamus. This process contributes to the central processing of orofacial pain perception in TN models. These findings offer novel insights on the mechanisms underlying TN and suggest that TNC astrocytes play a crucial role in trigeminal neuralgia therefore targeting astrocyte activity may hold promise as a therapeutic approach in reducing or preventing neuropathic pain associated with this condition.

### Supplementary Information

**Additional file 1: Figure S1.** A. Experimental timeline B. Schematic diagram showing the stereotaxic coordinates for optogenetic or null virus injection in a rat. C. Schematic diagram of rat brain showing optic stimulation and recording site. **Figure S2.** Immunofluorescent images showing the efficiency of AAV virus transfection. A. Schematic diagram from Paxinos rat brain atlas showing the coronal section of trigeminal spinal caudalis in rat brain and mCherry transfection in the region. B. Quantification of virus transfection (represented by mCherry fluorescence intensity) in different groups. *N*= 7/groupOrdinary one-way ANOVA, *p*=0.0744, not significant with other groups. Immunofluorescent images of mCherry transfection in © TN/eNpHR+ (D) TN/eNpHR- (E) sham/ eNpHR+ (F) sham/ eNpHR- . Scale = 50μm. **Figure S3.** Representative peri event spectrograms of TN/eNpHR+ rat in light off (A) and light on condition (B). Multiple linear regression graphs showing burst activity of TN/eNpHR+ rats in light off (C) and light on (D) conditions. **Figure S4.** C-fos expression in the TNC in response to astrocyte-specific TNC optogenetic inhibition. Representative immunohistochemistry images showing c-fos expression in the TNC of (A, C) TN/eNpHR+ (B, D) TN/eNpHR- animals in response to astrocyte-specific TNC optogenetic inhibition. (E) Quantification of c-fos positive cells in the TNC of TN rats with optogenetic inhibition with a yellow laser. Data are presented as the mean ± SD. Two-tailed Mann-Whitney test, *n*=6/group, **P=0.0022 compared to another group. **Figure S5.** CGRP and Iba1 immunoreactivity in TN rats in response to optic inhibition of TNC astrocytes. (A,B) Representative immunofluorescent images of CGRP receptor expression in trigeminal ganglion of TN/eNpHR+ and TN/ eNpHR- rats. Scale= 50μm (C-F) Representative immunofluorescent images of microglial (Iba1) expression in trigeminal nucleus caudalis of TN/eNpHR+ and TN/ eNpHR- rats. Scale =200μm and 50μm (G) Quantification of CGRP fluorescent intensity (H) Quantification of Iba1 fluorescent intensity. Data are presented as the mean ± SD. Two-tailed Mann-Whitney test, *n*=6/group, **P=0.0043 compared to another group. **Table S1.** Key resources.

## Data Availability

Data can be made available upon reasonable request to corresponding author.

## Abbreviations

AAV: Adeno-associated virus
AT: Acetone test
APT: Air puff test
CNS: Central nervous system
CGRP: Calcitonin gene-related peptide
DAPI: 4′,6-Diamidino-2-phenylindole
GFAP: Glial fibrillary acidic protein
IACUC: Institutional Animal Care and Use Committee
Iba1: Ionized calcium-binding adapter molecule 1
IONC: Infraorbital nerve chronic constriction
NpHR: Halorhodopsin from Natronomonas
OFT: Open field test
P2X3: P2X purinoceptor 3
PFA: Paraformaldehyde
TNC: Trigeminal nucleus caudalis
TG: Trigeminal ganglion
TN: Trigeminal neuralgia
VF: Von Frey filaments test
VPM: Ventral posteromedial nucleus of thalamus
VTT: Ventral trigeminothalamic tract
