# Supplementary material for: GFAP-NpHR mediated optogenetic inhibition of trigeminal nucleus caudalis attenuates hypersensitive behaviors and thalamic discharge attributed to infraorbital nerve constriction injury
Source: J Headache Pain. 2023 Oct 11;24(1):137. doi: 10.1186/s10194-023-01669-z (PMC10566148; doi:10.1186/s10194-023-01669-z)
Supplement: Supplementary file 1 — Additional file 1: Figure S1. A. Experimental timeline B. Schematic diagram showing the stereotaxic coordinates for optogenetic or null virus injection in a rat. C. Schematic diagram of rat brain showing optic stimulation and recording site. Figure S2. Immunofluorescent images showing the efficiency of AAV virus transfection. A. Schematic diagram from Paxinos rat brain atlas showing the coronal section of trigeminal spinal caudalis in rat brain and mCherry transfection in the region. B. Quantification of virus transfection (represented by mCherry fluorescence intensity) in different groups. N= 7/groupOrdinary one-way ANOVA, p=0.0744, not significant with other groups. Immunofluorescent images of mCherry transfection in © TN/eNpHR+ (D) TN/eNpHR- (E) sham/ eNpHR+ (F) sham/ eNpHR- . Scale = 50μm. Figure S3. Representative peri event spectrograms of TN/eNpHR+ rat in light off (A) and light on condition (B). Multiple linear regression graphs showing burst activity of TN/eNpHR+ rats in light off (C) and light on (D) conditions. Figure S4. C-fos expression in the TNC in response to astrocyte-specific TNC optogenetic inhibition. Representative immunohistochemistry images showing c-fos expression in the TNC of (A, C) TN/eNpHR+ (B, D) TN/eNpHR- animals in response to astrocyte-specific TNC optogenetic inhibition. (E) Quantification of c-fos positive cells in the TNC of TN rats with optogenetic inhibition with a yellow laser. Data are presented as the mean ± SD. Two-tailed Mann-Whitney test, n=6/group, **P=0.0022 compared to another group. Figure S5. CGRP and Iba1 immunoreactivity in TN rats in response to optic inhibition of TNC astrocytes. (A,B) Representative immunofluorescent images of CGRP receptor expression in trigeminal ganglion of TN/eNpHR+ and TN/ eNpHR- rats. Scale= 50μm (C-F) Representative immunofluorescent images of microglial (Iba1) expression in trigeminal nucleus caudalis of TN/eNpHR+ and TN/ eNpHR- rats. Scale =200μm and 50μm (G) Quantification of CGRP fluore [file 10194_2023_1669_MOESM1_ESM.pdf]

Additional file 1

GFAP-NpHR mediated optogenetic inhibition of trigeminal nucleus caudalis attenuates hypersensitive behaviors and thalamic discharge attributed to infraorbital nerve constriction injury

Elina KC[1], Jaisan Islam[1], Hyong Kyu Kim[2], Young Seok Park[1,3*]

Author affiliations:

[1]Graduate Program in Neuroscience, Department of Medicine, College of Medicine, Chungbuk National University, Cheongju 28644, Republic of Korea

[2]Department of Medicine and Microbiology, College of Medicine, Chungbuk National University, Cheongju 28644, Republic of Korea

[3]Department of Neurosurgery, Chungbuk National University Hospital, Cheongju 28644, Republic of Korea

Corresponding author:

Young Seok Park, MD, PhD

Professor, Dept. of Neurosurgery, Chungbuk National University Hospital

College of Medicine, Chungbuk National University, Cheongju-Si, Chungbuk, Korea

Address: 776, 1 Sunhwanro, Seowon-gu, Cheongju-si, Korea Zip: 28644

Tel: +82-432696080, +82-1054188453 Fax: +82432731614

E-mail: youngseokparkmd@gmail.com, radiosurgerypark@chungbuk.ac.kr

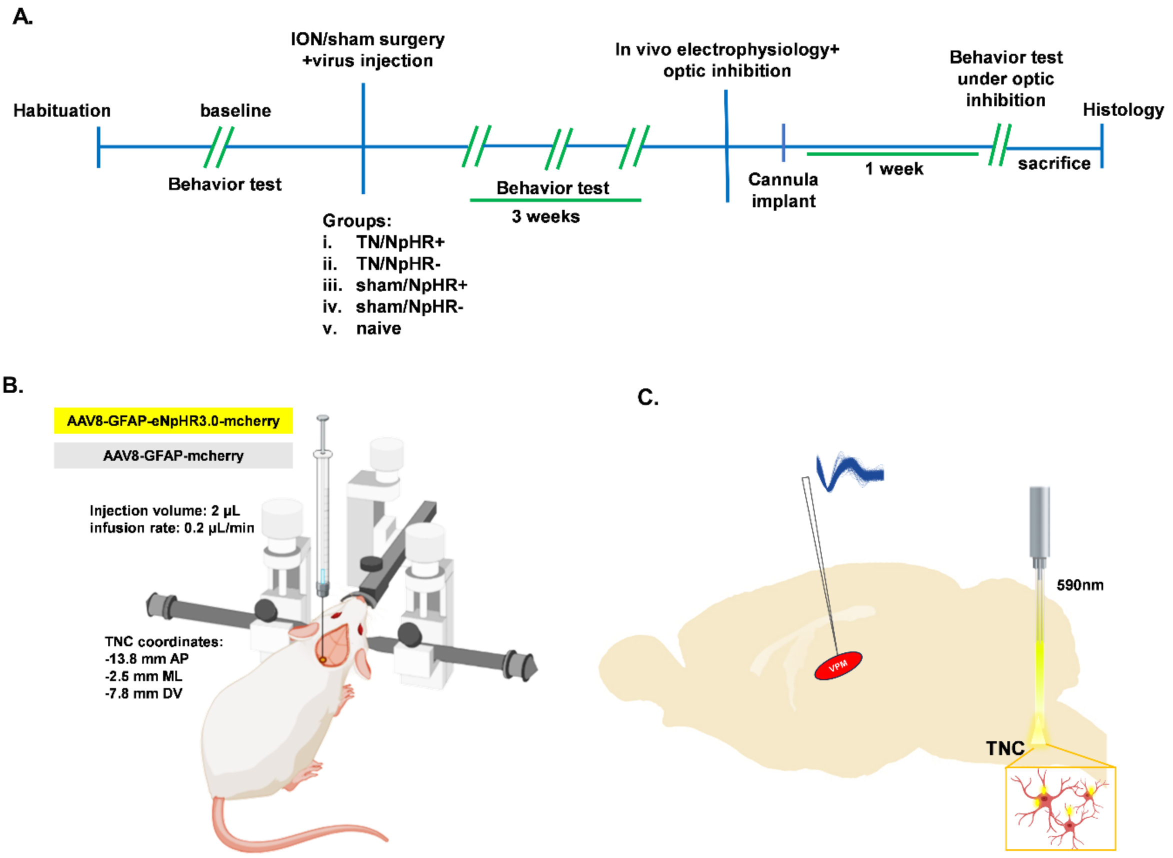

Figure S1. A. Experimental timeline B. Schematic diagram showing the stereotaxic coordinates for optogenetic or null virus injection in a rat. C. Schematic diagram of rat brain showing optic stimulation and recording site.

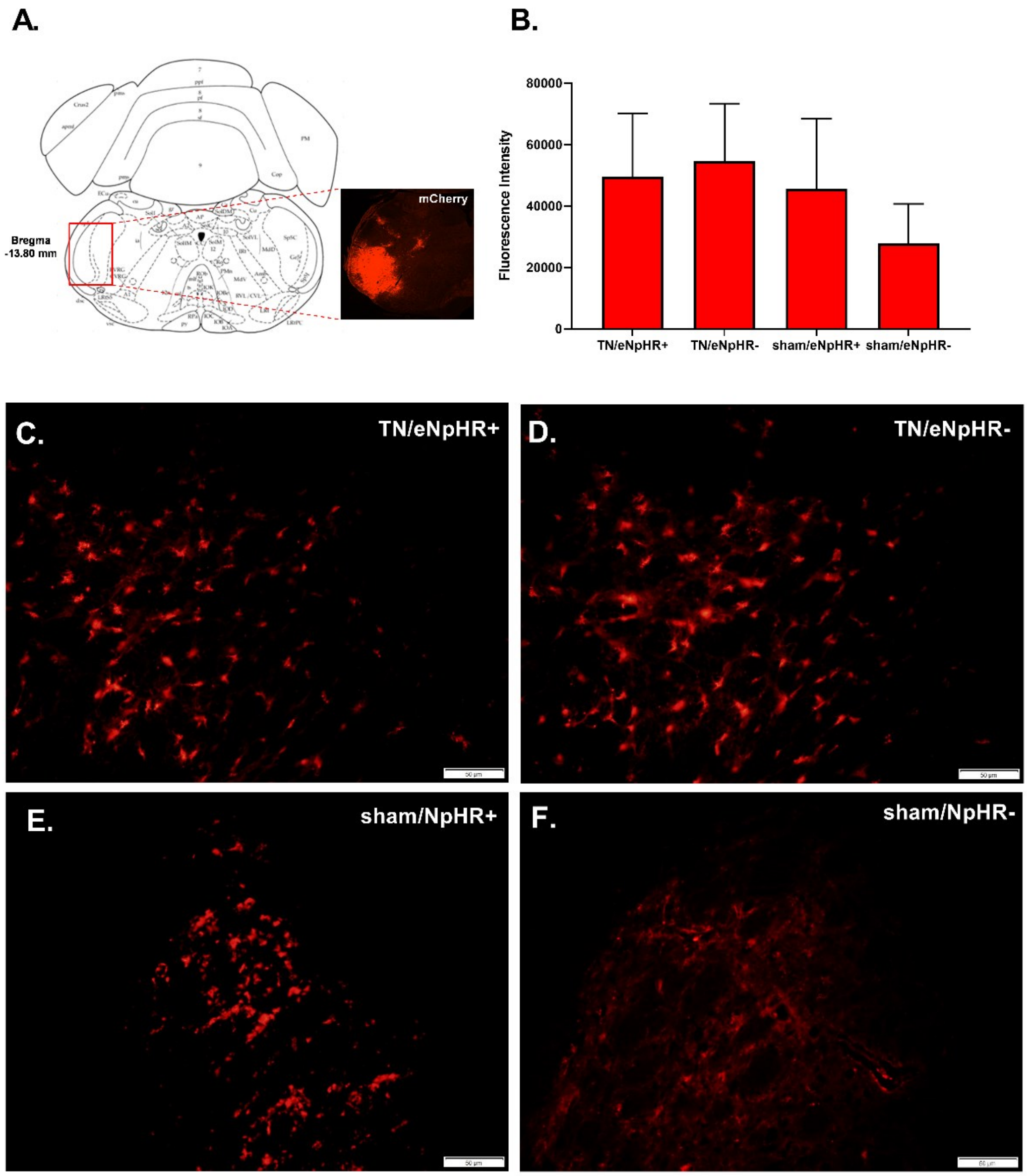

Figure S2. Immunofluorescent images showing the efficiency of AAV virus transfection. A. Schematic diagram from Paxinos rat brain atlas showing the coronal section of trigeminal spinal caudalis in rat brain and mCherry transfection in the region. B. Quantification of virus transfection (represented by mCherry fluorescence intensity) in different groups. N= 7/groupOrdinary one-way ANOVA, p=0.0744, not significant with other groups. Immunofluorescent images of mCherry transfection in © TN/eNpHR+ (D) TN/eNpHR- (E) sham/ eNpHR+ (F) sham/ eNpHR- . Scale = 50μm.

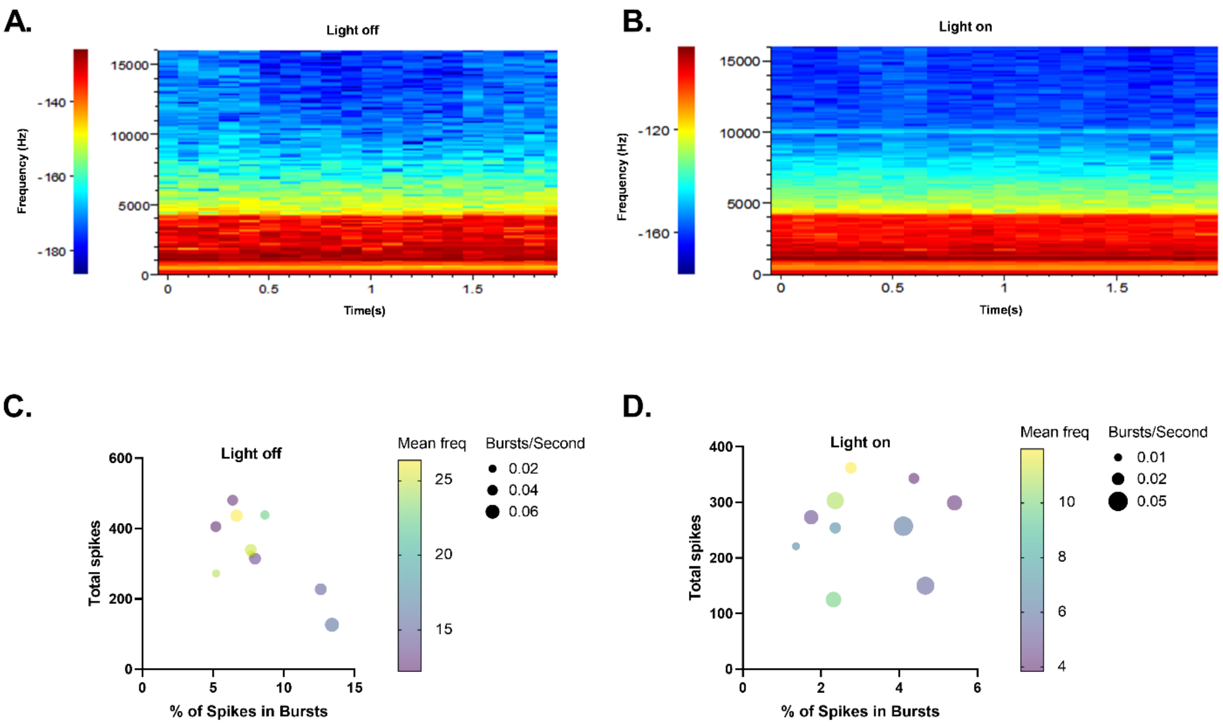

Figure S3. Representative peri event spectrograms of TN/eNpHR+ rat in light off (A) and light on condition (B). Multiple linear regression graphs showing burst activity of TN/eNpHR+ rats in light off (C) and light on (D) conditions.

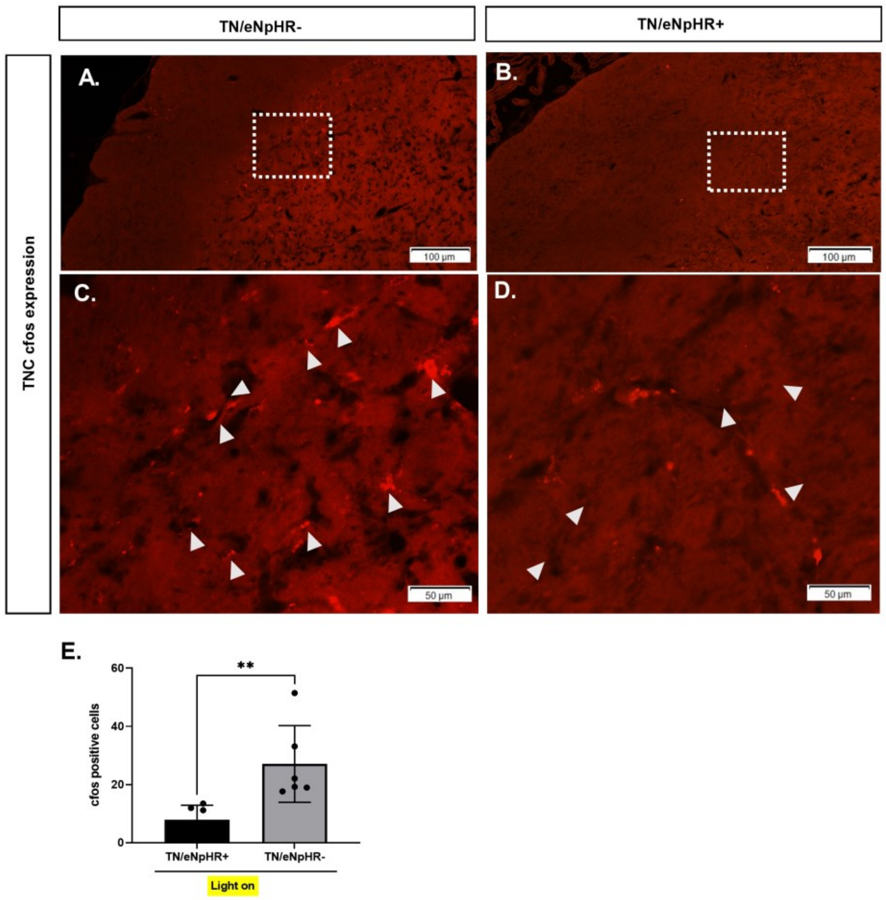

Figure S4. C-fos expression in the TNC in response to astrocyte-specific TNC optogenetic inhibition. Representative immunohistochemistry images showing c-fos expression in the TNC of (A, C) TN/eNpHR+ (B, D) TN/eNpHR- animals in response to astrocyte-specific TNC optogenetic inhibition. (E) Quantification of c-fos positive cells in the TNC of TN rats with optogenetic inhibition with a yellow laser. Data are presented as the mean ± SD. Two-tailed Mann-Whitney test, n=6/group, **P=0.0022 compared to another group.

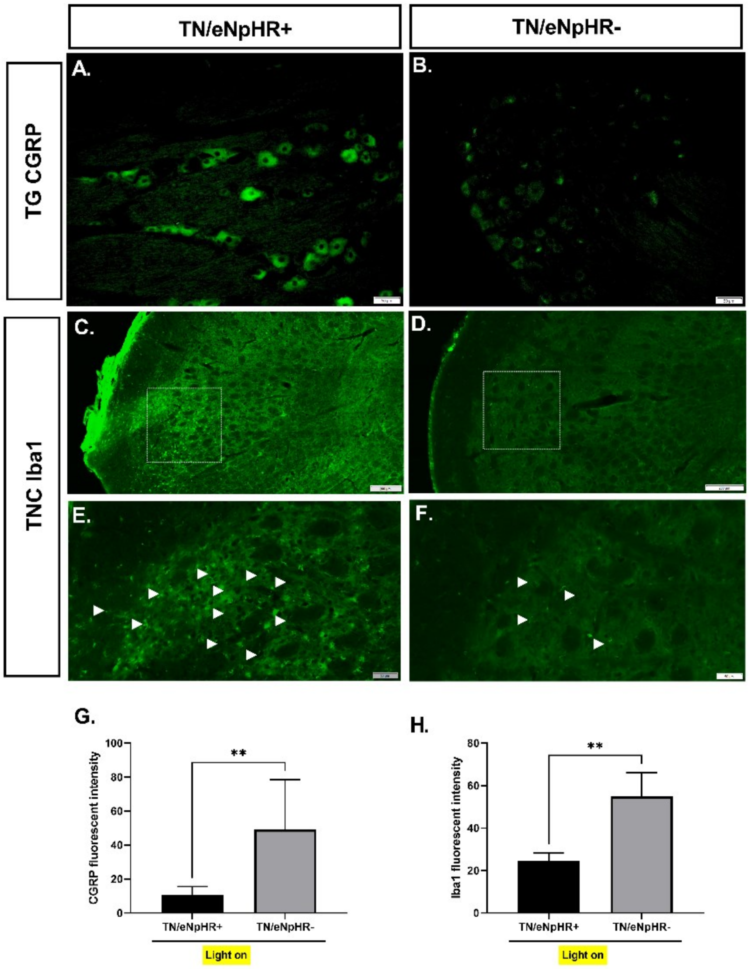

Figure S5. CGRP and Iba1 immunoreactivity in TN rats in response to optic inhibition of TNC astrocytes. (A,B) Representative immunofluorescent images of CGRP receptor expression in trigeminal ganglion of TN/eNpHR+ and TN/ eNpHR- rats. Scale= 50µm (C-F) Representative immunofluorescent images of microglial (Iba1) expression in trigeminal nucleus caudalis of TN/eNpHR+ and TN/ eNpHR- rats. Scale =200µm and 50µm (G) Quantification of CGRP fluorescent intensity (H) Quantification of Iba1 fluorescent intensity. Data are presented as the mean ± SD. Two-tailed Mann-Whitney test, n=6/group, **P=0.0043 compared to another group.

Table S1. Key resources

REAGENT or RESOURCE	SOURCE	IDENTIFIER
**Antibodies**		
Recombinant rabbit monoclonal anti-GFAP	Abcam	Cat# ab68428, RRID:AB_1209224
Mouse monoclonal ant- c-fos	Abcam	Cat# ab208942, RRID:AB_2747772
Recombinant mouse monoclonal anti-Iba1	Abcam	Cat# ab283319, RRID:AB_2924797
mouse monoclonal anti-CGRP	Abcam	Cat# ab81887, RRID:AB_1658411
Goat Anti-Rabbit IgG H&L (Alexa Fluor 488)	Abcam	Cat# ab150077, RRID:AB_2630356
Goat Anti-Mouse IgG H&L (Alexa Fluor 488)	Abcam	Abcam Cat# ab150113, RRID:AB_2576208
Recombinant anti-P2X3 antibody	Abcam	Cat# ab300493, RRID:AB_2941895
Biotinylated Anti-Mouse/Rabbit IgG (Universal)	Vector Laboratories	Cat# BP-1400-50, RRID:AB_2910231
**Bacterial and virus strains**		
AAV8-GFAP-eNpHR3.0-mcherry	Virus Facility, Korea Institute of Science and Technology	N/A
AAV8-GFAP-mcherry	Virus Facility, Korea Institute of Science and Technology	N/A
**Experimental models: Organisms / Strains**		
Sprague Dawley rat	Koatech Bio	RRID:RGD_5508397
**Software and algorithms**		
GraphPad Prism 9	GraphPad Software	RRID:SCR_002798
Cheetah Acquisition System	Neuralynx	https://neuralynx.com/software/cheetah
SpikeSort 3D	Neuralynx	RRID:SCR_014478
NeuroExplorer version 5	Nex Technologies	RRID:SCR_001818
ToxTrac v2.61	SourceForge	RRID:SCR_021502
ImageJ	National Institutes of Health	RRID:SCR_003070
cellSens Standard	Olympus	RRID:SCR_014551
OlyVia 2.4	Olympus	RRID:SCR_016167
G*Power version 3.1.9.4	Heinrich-Heine-Universität Düsseldorf	RRID:SCR_013726

**Others**		
Fiber optic cannula	Doric Lenses	MFC_200/230-0.48_###_ZF2.5_A45
Microelectrode	Kation Scientific	E1011-20, Carbostar-1
Electronic interface board	Neuralynx	EIB-36
589-nm yellow DPSS laser	Shanghai Laser & Optics Century Co., Ltd	YL589T3-010FC
VECTASTAIN Elite ABC-Peroxidase Kit	Vector Laboratories	Cat# PK-7200, RRID:AB_2336828
DAB substrate kit	Vector Laboratories	Cat# SK-4100, RRID:AB_2336382
Automated microsyringe pump	Harvard Apparatus	KD Scientific Legato® 130 Syringe Pump
Pneumatic pump module	Harvard Apparatus	BH2 system
